# Suppression of innate immunity by the vaccinia virus protein N1 promotes skin microbiota expansion and increased immune infiltration following vaccination

**DOI:** 10.1099/jgv.0.001814

**Published:** 2022-11-01

**Authors:** Evgeniya V. Shmeleva, Danial Syafiq, Ana L. Moldoveanu, Brian J. Ferguson, Geoffrey L. Smith

**Affiliations:** 1 Department of Pathology, University of Cambridge, Cambridge, UK

**Keywords:** innate immunity, N1, NF-κB, skin microbiota, Vaccinia virus

## Abstract

Vaccinia virus (VACV) protein N1 is an intracellular immunomodulator that contributes to virus virulence via inhibition of NF-κB. Intradermal infection with a VACV lacking gene *N1L* (vΔN1) results in smaller skin lesions than infection with wild-type virus (WT VACV), but the impact of N1 deletion on the local microbiota as well as the innate and cellular immune responses in infected ear tissue is mostly uncharacterized. Here, we analysed the bacterial burden and host immune response at the site of infection and report that the presence of protein N1 correlated with enhanced expansion of skin microbiota, even before lesion development. Furthermore, early after infection (days 1–3), prior to lesion development, the levels of inflammatory mediators were higher in vΔN1-infected tissue compared to WT VACV infection. In contrast, infiltration of ear tissue with myeloid and lymphoid cells was greater after WT VACV infection and there was significantly greater secondary bacterial infection that correlated with greater lesion size. We conclude that a more robust innate immune response to vΔN1 infection leads to better control of virus replication, less bacterial growth and hence an overall reduction of tissue damage and lesion size. This analysis shows the potent impact of a single viral immunomodulator on the host immune response and the pathophysiology of VACV infection in the skin.

Vaccinia virus (VACV) was used as the live vaccine to eradicate smallpox [1] and is the prototypal member of the genus *Orthopoxvirus* of the *Poxviridae* [2]. These large, double-stranded (ds)DNA viruses replicate in the cytoplasm and encode scores of proteins that modulate the host response to infection [3]. Many of these immunomodulatory proteins function intracellularly to suppress innate immune and programmed cell death signalling pathways, whilst others are secreted from infected cells, where they bind and neutralize interferons (IFNs), cytokines, chemokines and components of the complement system [4].

VACV protein N1 is a small, intracellular [5], Bcl-2-like [6, 7] protein that does not affect virus replication in cell culture [5], but blocks both NF-κB activation [8, 9] and apoptosis [7, 10] and contributes to virulence [5, 11]. Intradermal (i.d.) infection with a VACV strain Western Reserve (WR) mutant lacking the *N1L* gene (vΔN1) resulted in the same level of virus replication and virus antigen in infected tissue for the first 2 days of infection, but from day 3 onwards there were lower viral titres than for wild-type (WT) VACV [5, 10, 12]. vΔN1 also induced significantly smaller skin lesions [5, 10, 12]. Mutagenesis of N1 showed that N1 enhanced virulence solely via its ability to inhibit NF-κB [10]. The VACV N1 protein was reported to diminish the natural killer cell and VACV-specific CD8^+^ T cell response to infection [12, 13], and the corresponding protein from ectromelia virus also promoted virus virulence and increased viral loads in infected tissue [14]. Pathological changes that occur during viral infection are not only caused by virus–host interactions, and the commensal microbiota also contribute to pathogenesis and the outcomes of viral infections [15, 16]. Recently, dermal infection with VACV was shown to induce a large expansion of local skin microbiota that promote pathology and influence the immune response to infection [17]. Here, we investigated the local immune responses to i.d. infection of the mouse ear pinnae with vΔN1 or WT VACV to understand how inhibition of NF-κB by N1 affects the outcome of infection *in vivo* and to find factors responsible for the alteration in dermal lesion size.

To do this, C57BL/6 mice were injected i.d. in the ear pinnae with 10^4^ plaque-forming units (p.f.u.) of VΔΝ1 or its parental WT VACV [5]. Viral inocula used for injections were back-titrated by plaque assay and this confirmed that equal doses were administered. As noted previously, vΔN1 infection induced smaller skin lesions from day 6 post-injection (p.i.) (Fig. 1a) and lower viral titres from day 3 p.i. (Fig. 1b) compared to WT [5, 10, 12]. Since N1 does not affect the yields of intracellular and extracellular virus produced in cell culture [5], or viral titres on days 1 and 2 after i.d. infection *in vivo* [5, 10, 12], the difference in viral titres between these groups can be attributed to N1-mediated differences in the host immune responses to infection.

Our previous findings demonstrated that i.d. infection with VACV causes a large expansion of skin commensal bacteria, which promote tissue damage and lesion development, and significantly increases the recruitment of different leucocyte subpopulations, especially neutrophils, into the site of infection, without influencing viral titres [17]. To determine whether the N1 protein affected bacterial expansion, we counted bacterial colony-forming units (c.f.u.) grown on blood agar after seeding homogenates of vΔN1- or WT-VACV-infected ear tissues as described elsewhere [17]. Notably, on all four times p.i. tested, infection with vΔN1 induced significantly lower bacterial growth compared to WT virus (Fig. 1c). This might be due to the fact that vΔN1 is less suppressive of the early innate immune response, and therefore less able to create a favourable environment for the expansion of opportunistic microbiota. In turn, the lower bacterial expansion correlates with the decreased lesion sizes observed after vΔN1 infection. Indeed the changes in microbiota (day 3 p.i.) precede the start of measurable ear pathology (day 4 p.i.), and are therefore consistent with bacteria driving the ear pathology [17] and this response being reduced following infection with vΔN1.

To understand if the early inflammatory response following WT or vΔN1 infection is consistent with this model, ear pinnae were obtained from groups of animals at different times after i.d. infection, tissues were homogenized and cytokine concentrations were determined using a Magnetic Luminex Mouse Premixed Multy-Analyte kit (R and D Systems). A broad range of cytokines and chemokines were found in the tissues during infection (Fig. 2). At days 1 and 2 p.i. the majority of cytokines/chemokines analysed were similar to baseline levels before infection, as well as to levels in mock-infected tissue on days 1 and 3 post-injection of phosphate-buffered saline (PBS; data not shown). Most cytokine and chemokine levels started increasing to measurable levels at day 3 p.i., prior to observable pathological changes and reached a peak on days 5–6 (Fig. 2). Notably, at day 3 p.i. levels of IFNγ, IL-6, IL-12p70, CCL2, CCL4, CCL5 and CXCL10 were higher in ear pinnae infected with vΔN1 compared to WT, consistent with vΔN1 VACV being less able to inhibit the production of NF-κB-dependent inflammatory mediators . The production of these inflammatory mediators was delayed rather than ablated in the WT VACV group, although the maximum levels obtained were generally higher following infection by WT VACV. This delay in the initiation of innate immune response following WT infection likely plays a pivotal role in the failure to efficiently control WT viral replication, which leads to higher viral titres and greater expansion of commensal skin bacteria at the site of infection. A possible explanation for the reduction of lesion sizes observed after vΔN1 infection is that loss of N1 makes VACV less virulent because infected cells activate NF-κB more efficiently. Consequently, the host immune system responds faster to produce cytokines and chemokines, which help limit viral replication sooner and enables better control of skin microbiota than in case of WT VACV infection.

Next, we investigated the leukocytes recruited into the infected ear tissue by flow cytometry. The ear pinnae were collected at 3, 5, 7 and 9 days p.i., and ear tissue was incubated for 1 h in medium containing 750 U ml^-1^ of collagenase I and 100 U ml^-1^ of DNase I. Thereafter, leukocytes were extracted by centrifugation in 35% (v/v) isotonic Percoll. After intracellular staining with monoclonal antibodies (mAbs), cells were analysed on a BD LSRFortessa using gating strategies (Figs S1 and S2, available in the online version of this article). These data demonstrate that multiple subpopulations of leukocytes were significantly lower after vΔN1-infection compared to WT at all times analysed (Fig. 3). Interestingly, the recruitment of myeloid cells (particularly eosinophils, inflammatory Ly6C+monocytes and neutrophils) at day 3 p.i. did not correlate with levels of cytokines and chemokines in ear tissue, which were higher in the vΔN1 group, whilst cell recruitment was more prominent following WT VACV infection.

This may be due to greater levels of tissue damage and DAMP/alarmin release after infection with WT VACV, which may be a consequence of greater cytolysis of VACV-infected cells or greater levels of bacterial expansion at the site of infection. DAMPs are known to induce chemotaxis of immune cells, especially neutrophils, monocytes and macrophages, towards injured tissue [18–20], and this might explain the greater cell migration despite lower levels of cytokines and chemokines. At later time points, the number of cells recruited in ear tissue correlated positively with cytokine/chemokine levels and was significantly elevated in the WT VACV group. As suggested in our recent publication [17], expanded opportunistic bacteria from the skin surface invade the skin layers through VACV-mediated initial tissue damage, leading to bacterial-driven amplification of inflammation and immune cell infiltration (especially neutrophils) at a later time point. Recruited leukocytes secrete more pro-inflammatory cytokines and chemokines, which results in further attraction of immune cells to promote inflammation and clearance of virus. Notably, the absolute numbers of leukocytes recruited into the site of inflammation correlated with lesion sizes, and the strongest correlations were observed for neutrophil (*r*=0.733, *P*<0.0001) and CD8^+^ T cell (*r*=0.579, *P*=0.001) counts. Neutrophils can induce tissue damage by releasing reactive oxygen species, neutrophil extracellular traps and azurophil granules [21–23]. Further, CD8^+^ T cells are known for their cytotoxic function and ability to promote immunopathology [24].

Tissue damage during viral infection is a consequence of both virus replication and the normal immune reaction to pathogen invasion, and is an important factor for the development of adaptive immune response [25]. Tissue damage can also result from virus-induced expansion of commensal bacteria and be influenced by virus virulence and the early immune response. Therefore, elevated bacterial growth, increased numbers of immune cells presented in ear tissue and high levels of cytokine/chemokine production at later time points after i.d. infection with WT VACV can be explained by inefficient viral clearance. In turn, this might be explained by the delayed innate immune response to infection due to the ability of N1 to inhibit NF-κB activation. Previously, it was shown that the ability of N1 to inhibit NF-κB activation, rather than apoptosis, contributed to virus virulence and influenced the immune response to infection [10, 12].

In this study, we have shown that the attenuation of VACV by deletion of a single immunomodulatory protein, N1, has a profound effect on pathophysiology of skin infection and results in greater secretion of NF-κB-driven cytokines and chemokines at the early stage of infection, an increased ability of the host to control viral replication and growth of opportunistic bacteria, and ultimately reduced tissue damage.

## Supplementary Material

Supplementary figures

## Figures and Tables

**Fig. 1:**
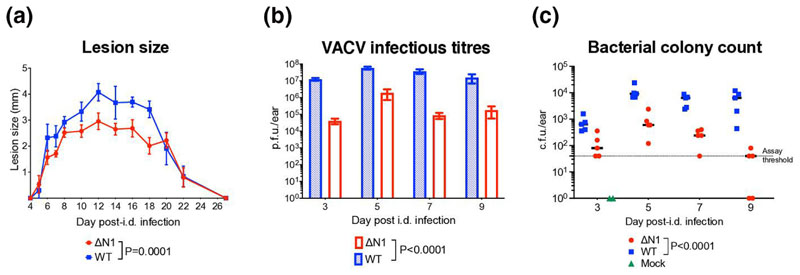
Dermal lesion sizes (a) and VACV infectious titres (b) of ear tissue at different times after i.d. infection with WT or vΔN1 VACV. Means with SEM are shown. *P* values were determined by the two-way ANOVA test. *n*=5 animals/group. (c) Bacterial colony-forming unit (c.f.u.) counts of WT- or vΔN1- or mock-infected ear samples collected at different times p.i. Medians are shown. Statistical analysis was by the RM ANOVA test. Experiments were performed twice and data shown are representative of both experiments.

**Fig. 2:**
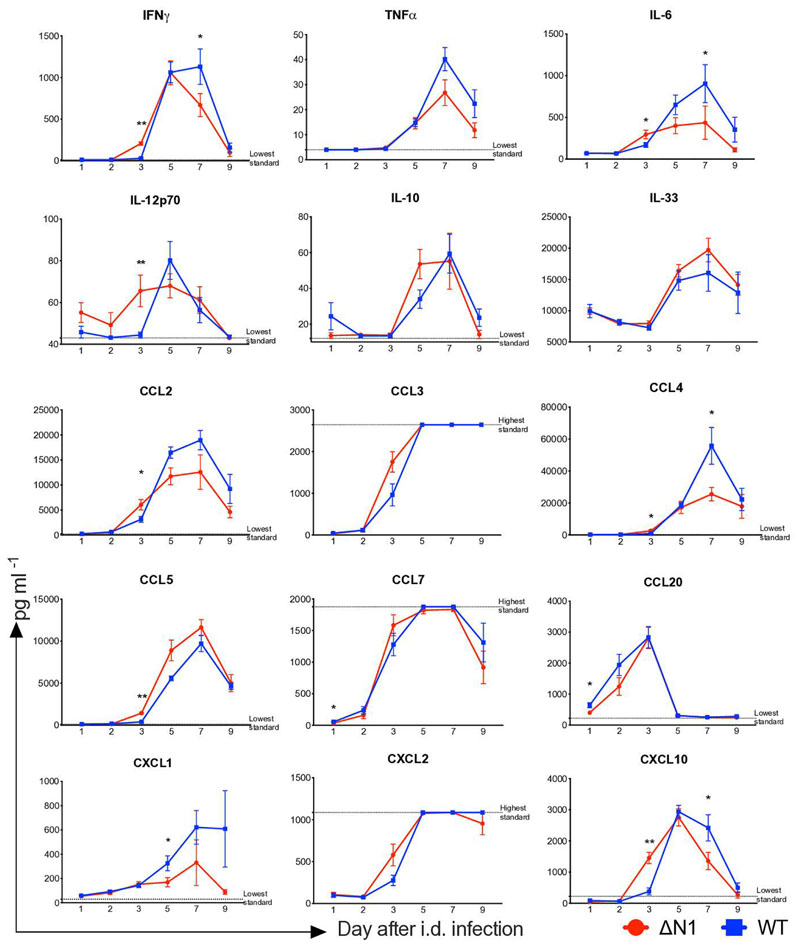
Levels of cytokines and chemokines detected by multiplex assay (Luminex) in ear tissues at different times after i.d. infection with vΔA1 or WT VACV. Means with SEM are shown. *P* values were determined by the Mann–Whitney test. *, *P*<0.05; **, *P*<0.01. *n*=5 animals per group. This experiment was performed once.

**Fig. 3:**
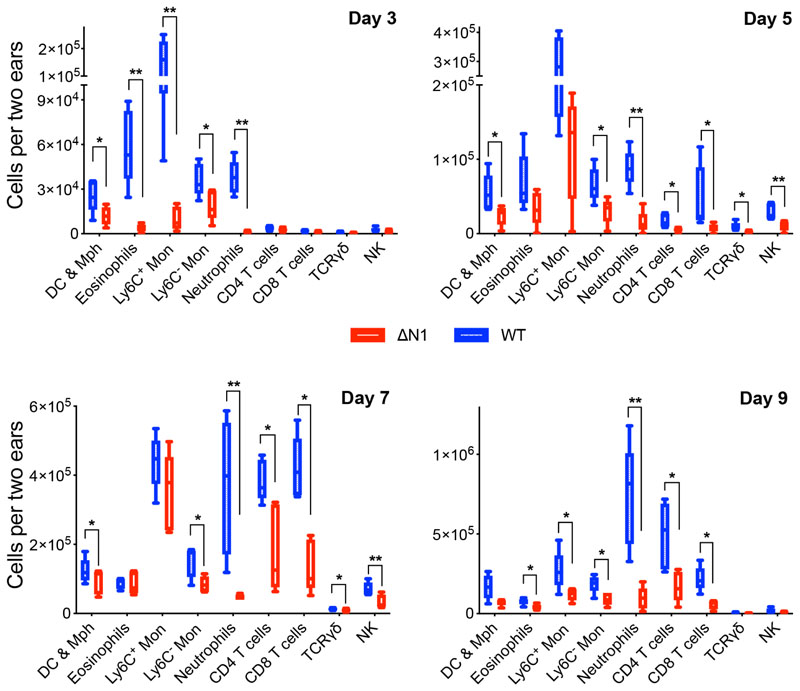
(a) Absolute counts of different subpoputations of leukocytes infiltrating ear tissue at 3, 5, 7 and 9 days p.i. with WT or vΔN1 VACV. Box plots are shown. *P* values were determined by the Mann–Whitney test. *, *P*<0.05; **, *P*<0.01. *n*=5 animals/group. Experiments were performed twice and data shown are representative of both experiments.
