# Supplementary material for: Suppression of innate immunity by the vaccinia virus protein N1 promotes skin microbiota expansion and increased immune infiltration following vaccination
Source: J Gen Virol. Author manuscript; Available in PMC 2023 Jul 31. (PMC7614846; doi:10.1099/jgv.0.001814)
Supplement: Supplementary figures [file EMS179319-supplement-Supplementary_figures.pdf]

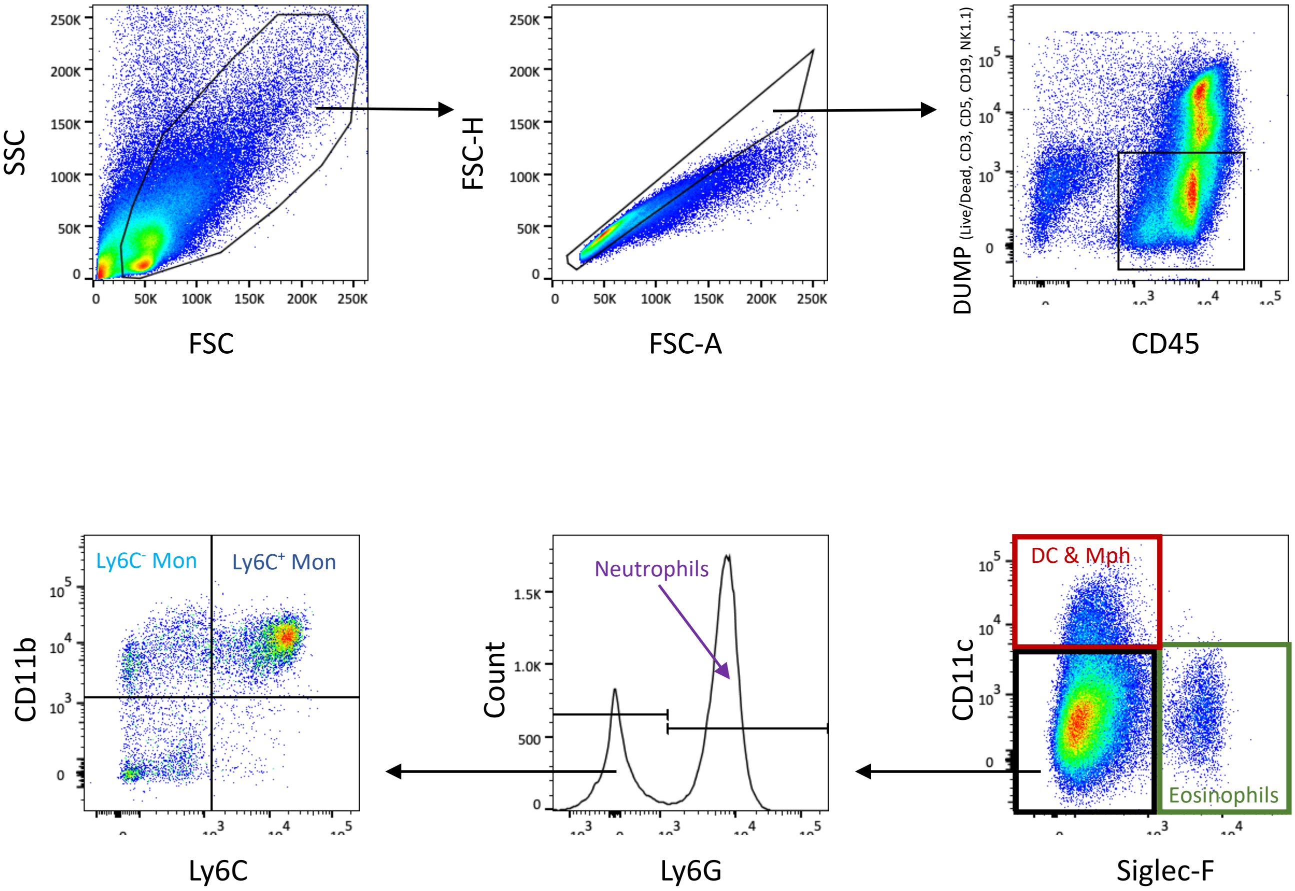

Supplementary Figure 1. Gating strategy for flow cytometry assay of myeloid lineage cells present in mouse ear tissue after intradermal injection with VACV. Cells were gated on their characteristic ability to scatter light. Doublets were excluded using FSC-A (area) versus FSC-H (height) plot. The myeloid gate included CD45^{+}Zombie dye^{-}CD3^{-}CD5^{-}CD19^{-}NK1.1^{-} cells. Further myeloid cell subpopulations were classified as the following phenotypes:

Eosinophils: CD45^{+}CD3^{-}CD5^{-}CD19^{-}NK1.1^{-}CD11c^{-}Siglec-F^{+};

DC and Mph (dendritic cells and macrophages): CD45^{+}CD3^{-}CD5^{-} CD19^{-}NK1.1^{-}Siglec-F^{-}CD11c^{+};

Neutrophils: CD45^{+}CD3^{-}CD5^{-}CD19^{-}NK1.1^{-}CD11c^{-}Siglec-F^{-} Ly6G^{+};

Ly6C^{+}Mon (inflammatory monocytes): CD45^{+}CD3^{-}CD5^{-}CD19^{-}NK1.1^{-}CD11c^{-}Siglec-F^{-}Ly6G^{-}CD11b^{+}Ly6C^{+};

Ly6C^{-}Mon (residential monocytes): CD45^{+}CD3^{-}CD5^{-}CD19^{-}NK1.1^{-}CD11c^{-}Siglec-F^{-}Ly6G^{-}CD11b^{+}Ly6C^{-}.

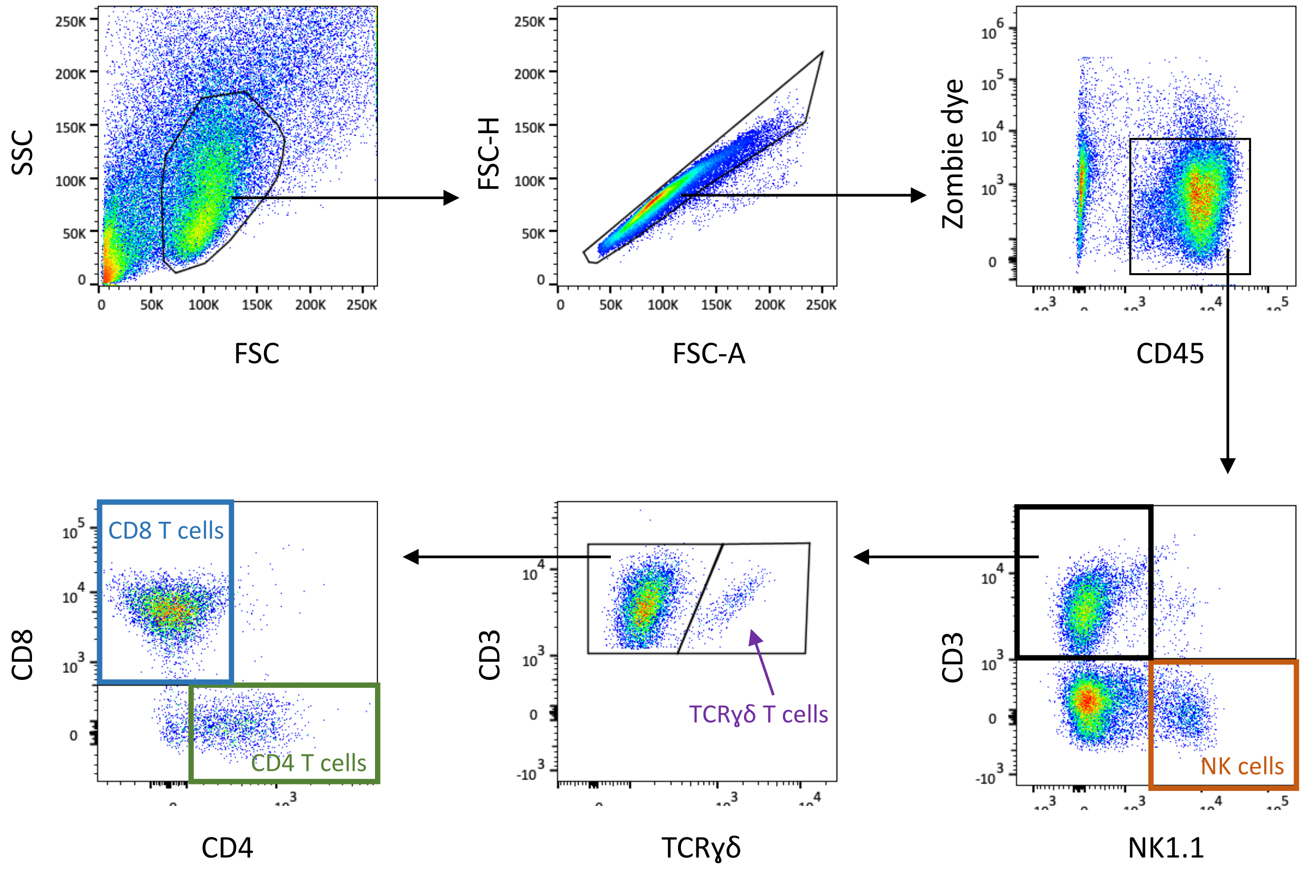

Supplementary Figure 2. Gating strategy for flow cytometry assay of lymphoid lineage cells present in mouse ear tissue after intradermal injection with VACV. Cells were gated on their characteristic ability to scatter light. Doublets were excluded using FSC-A (area) versus FSC-H (height) plot and then haemopoietic cells were gated as $CD45^{+}$Zombie dye^{-} cells. Further lymphoid subpopulations were classified as the following phenotypes:

Natural killer (NK) cells: $CD45^{+}CD3^{-}NK1.1^{+}$;

TCRγδ T cells: $CD45^{+}CD3^{+}NK1.1^{-}$ $TCR\gamma\delta^{+}$

CD4 T cells (TCRαβ): $CD45^{+}CD3^{+}NK1.1^{-}TCR\gamma\delta^{-}CD8^{-}CD4^{+}$;

CD8 T cells (TCRαβ): $CD45^{+}CD3^{+}NK1.1^{-}TCR\gamma\delta^{-}CD4^{-}CD8^{+}$.
